# Return of individual research results to participants with and at risk for Parkinson’s disease

**DOI:** 10.1017/cts.2024.616

**Published:** 2024-10-15

**Authors:** Samantha E. Lettenberger, Emily A. Hartman, Kali Tam, Peggy Auinger, Meghan E. Pawlik, Renee Wilson, Elizabeth T. Banda, Blanca Valdovinos, Daniel Kinel, Roy N. Alcalay, E. Ray Dorsey, Lucy Norcliffe-Kaufmann, Saloni Sharma, Robert G. Holloway, Ruth B. Schneider

**Affiliations:** 1 Center for Health + Technology, University of Rochester Medical Center, Rochester, NY, USA; 2 University of Rochester, Rochester, NY, USA; 3 Department of Neurology, University of Rochester Medical Center, Rochester, NY, USA; 4 Department of Neurology, Tel Aviv Sourasky Medical Center, Tel Aviv, Israel; 5 Department of Neurology, Columbia University Irving Medical Center, New York, NY, USA; 6 23andMe, Inc., Sunnyvale, CA, USA

**Keywords:** Lrrk2, Parkinson’s disease, research ethics, telemedicine, return of results, research dissemination

## Abstract

In a prospective, remote natural history study of 277 individuals with (60) and genetically at risk for (217) Parkinson’s disease (PD), we examined interest in the return of individual research results (IRRs) and compared characteristics of those who opted for versus against the return of IRRs. Most (*n* = 180, 65%) requested sharing of IRRs with either a primary care provider, neurologist, or themselves. Among individuals without PD, those who requested sharing of IRRs with a clinician reported more motor symptoms than those who did not request any sharing (mean (SD) 2.2 (4.0) versus 0.7 (1.5)). Participant interest in the return of IRRs is strong.

## Introduction

With increasing discussions regarding participants’ right to their own research data, there has been a growing demand for the return of individual research results (IRRs). The return of IRRs stands to benefit both the individual and the research team through increased understanding of personal health, increased awareness of health risks, increased trust in researchers, and increased likelihood of research participation [1,2].

However, when determining whether, what, how, when, and with whom to share IRRs in prospective research studies, we must carefully consider how this practice might impact both the individual and the study. For example, the return of IRRs, particularly when given without appropriate context, could cause unwarranted participant anxiety, lead to unnecessary medical evaluation [3], or cause distress to participants and their healthcare providers. The potential risks are greater when working with vulnerable research populations or returning sensitive health information, such as that pertaining to the risk of a neurodegenerative disorder. Additionally, the return of in-study findings in real-time could alter participant behavior, introduce bias or, in the case of clinical trials, result in inadvertent unblinding [4].

Such questions become even more challenging to answer when the population under study is one that is genetically at risk for the development of a progressive neurodegenerative disorder, such as Parkinson’s disease (PD). Carriers of the *LRRK2* G2019S variant, an autosomal dominant genetic cause of PD that exhibits reduced penetrance, are one such group. Little is known regarding interest, among individuals with and genetically at risk for PD, in the return of clinical results. In a remote natural history study, we are prospectively characterizing *LRRK2* G2019S carriers. Here, we describe our approach to the return of IRRs, report participant preferences regarding the return of IRRs, and compare characteristics of those who opted for versus against the return of IRRs.

## Materials and methods

### Study design

VALOR-PD is a remote, nationwide, natural history study of *LRRK2* G2019S carriers with and without PD. Participants were identified through collaboration with 23andMe, Inc., a direct-to-consumer genetic testing company, and were already aware of their genetic status at the time of initial study contact. The original aims of the study were to evaluate the feasibility of recruiting and retaining participants using this unique research model, to prospectively characterize the cohort and compare it to traditionally established *LRRK2* cohorts, and to assess the ability of this model to create a clinical trial-ready cohort. Participants in this ongoing study complete video-based visits, which include motor, sleep, autonomic, cognitive, and mood assessments, annually for 36 months. The assessments were selected to capture the broad range of motor and non-motor symptoms that are characteristic of PD. The study was approved by the University of Rochester’s Research Subjects Review Board (STUDY00003703), and all participants provided informed consent. Full descriptions of the study methodology [5] and baseline results [6] have been previously published.

### Return of individual research results

The VALOR-PD Steering Committee, which includes clinical researchers, an individual with PD, and an individual with a *LRRK2* variant, considered at the outset of the study whether and how to offer and provide IRRs. We developed the following processes and materials based on these discussions. During the informed consent process, participants were asked: “Would you like the study team to share a personalized summary of your research participation following each virtual research visit with your primary care provider?” and “Would you like the study team to share a personalized summary of your research participation following each virtual research visit with your neurologist?” We did not ask participants if they would like to personally receive their IRRs given concern that this might cause unwarranted anxiety or distress among participants genetically at risk for PD. However, we anticipated this request and decided at the outset of the study to provide them if requested. Results were typically shared within 30 days of the participant’s annual visit via posted mail. Receipt of IRRs was not verified by the study team, and information on how participants’ medical teams used IRRs was not obtained.

**Figure 1.**
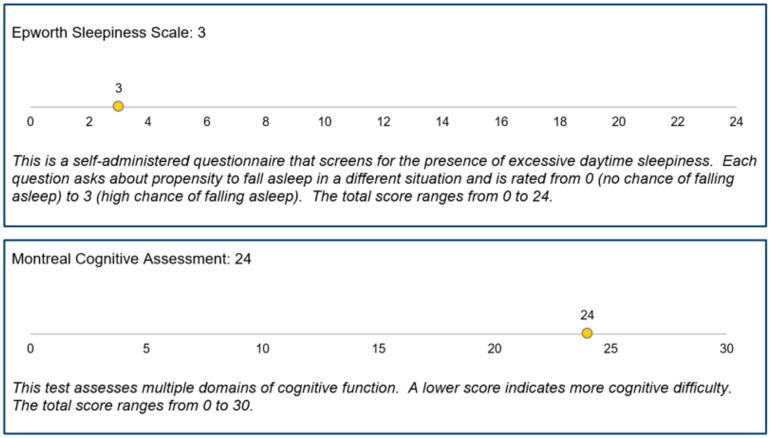
Examples of information included in the individual research report.

The IRRs summary provides scores for select assessments – the Movement Disorders Society – Unified Parkinson’s Disease Rating Scale (MDS-UPDRS) Part III Motor Examination (modified for remote examination), REM Sleep Behavior Disorder Screening Questionnaire, Epworth Sleepiness Scale, Beck Depression Inventory-II (BDI-II), and Montreal Cognitive Assessment – completed after each participant’s video-based research visit (Figure 1). For each assessment, the IRR summary also includes a brief description and general information about scoring. The IRR summary does not include any specific follow-up or testing recommendations. Contact information for the study’s principal investigator is included in all IRRs. Contact information for the National Suicide Prevention Lifeline is included when a participant scores ≥14 on the BDI-II or ≥1 on the BDI-II Suicidal Thoughts or Wishes item.

### Statistical analyses

We used descriptive statistics to summarize rates of the requested return of IRRs. To examine differences among those who requested the return of IRRs to themselves, their primary care provider (PCP) or neurologist, themselves and a clinician, and those who did not request the return of IRRs, we compared baseline demographic and clinical characteristics among those with and without PD using analysis of variance for continuous characteristics and Chi-square or Fisher’s exact tests for categorical characteristics. Pairwise group comparisons were performed using the Tukey–Kramer test for analysis of variance to control the type I error probability. P-values <0.05 were considered statistically significant. No adjustment was made for comparisons of multiple characteristics.

## Results

VALOR-PD enrolled 277 participants from 34 states. At baseline, 60 participants had self-reported PD (mean [SD] age 67.8 years [8.4], 98% white, 52% female, 98% greater than high school education, 80% Ashkenazi Jewish, and 67% with a family history of PD), and 217 did not have self-reported PD (53.7 years [15.1], 95% white, 59% female, 99% greater than high school education, 73% Ashkenazi Jewish, and 57% with a family history of PD) [6].

In total, 180 (65%) participants (52/60 with PD, 128/217 without PD) requested sharing of IRRs with a PCP, neurologist, or with them personally. Seventy-seven (28%) participants (20/60 with PD, 57/217 without PD) requested sharing of IRRs with their PCP, 55 (20%) participants (42/60 with PD, 13/217 without PD) with their neurologist, and 108 (39%) participants (23/60 with PD, 85/217 without PD) with them personally. Of those who requested the return of IRRs, 54 (30%) participants (31/52 with PD, 23/128 without PD) requested sharing with more than one individual.

As seen in Table 1, among *LRRK2* carriers with PD, there was an overall statistical difference in mean age at baseline (*p* = 0.02) though no individual pairwise comparison was significant. Among *LRRK2* carriers without PD, individuals who requested sharing of IRRs with a clinician had higher MDS-UPDRS part II scores than those who did not (mean [SD] 2.2 [4.0] versus 0.7 [1.5], *p* 0.01).

**Table 1.** Baseline characteristics of the study cohort by status of requesting individual research results

	*LRRK2* carriers with PD (*n* = 60)					*LRRK2* carriers without PD (*n* = 217)				
	Personal only* (*n* = 7)	Clinician only (*n* = 29)	Both (*n* = 16)	None (*n* = 8)	*P* value	Personal only* (*n* = 68)	Clinician only (*n* = 43)	Both (*n* = 17)	None (*n* = 89)	*P* value
Age, years	61.6 (11.2)	69.9 (7.6)	69.3 (5.2)	62.4 (10.0)	0.02	54.0 (14.8)	54.8 (14.0)	61.5 (13.5)	51.4 (15.8)	0.08
Women, n (%)	2 (28.6)	14 (48.3)	9 (56.3)	6 (75.0)	0.34	39 (57.4)	23 (53.5)	9 (52.9)	57 (64.0)	0.61
White race, n (%)	6 (85.7)	29 (100.0)	16 (100.0)	8 (100.0)	0.12	67 (98.5)	39 (90.7)	17 (100.0)	82 (92.1)	0.15
Hispanic/Latino, n (%)	0 (0.0)	2 (6.9)	1 (6.3)	1 (12.5)	0.88	4 (5.9)	3 (7.0)	0 (0.0)	9 (10.1)	0.58
Ashkenazi Jewish, n (%)	7 (100.0)	23 (79.3)	12 (75.0)	6 (75.0)	0.61	50 (73.5)	30 (69.8)	14 (82.4)	65 (73.0)	0.80
> High school education, n (%)	7 (100.0)	28 (96.6)	16 (100.0)	8 (100.0)	0.99	66 (97.1)	43 (100.0)	17 (100.0)	88 (98.9)	0.69
Family history of PD, n (%)	5 (71.4)	18 (62.1)	11 (68.8)	6 (75.0)	0.93	38 (55.9)	22 (51.2)	10 (58.2)	54 (60.7)	0.77
New to PD or *LRRK2* research participation, n (%) (*n* = 263)	2 (28.6)	8 (28.6)	10 (62.5)	1 (12.5)	0.06	48 (72.7)	36 (90.0)	12 (75.0)	63 (76.8)	0.21
MDS-UPDRS										
Part I	11.9 (6.1)	11.8 (5.0)	9.7 (6.0)	9.5 (4.6)	0.51	5.4 (4.0)	7.1 (4.9)	7.2 (8.3)	6.0 (5.0)	0.27
Part II (*n* = 274)	10.3 (8.6)	11.1 (7.5)	9.9 (8.7)	8.9 (7.5)	0.90	1.0 (2.1)	2.2 (4.0)^a^	1.4 (3.0)	0.7 (1.5)^a^	0.01
Part III (modified)	24.7 (8.4)	24.0 (11.0)	20.6 (10.3)	18.0 (14.1)	0.46	1.9 (4.0)	2.1 (4.0)	2.2 (3.8)	1.1 (2.8)	0.32
MoCA	27.4 (1.9)	26.8 (2.1)	27.6 (1.7)	26.6 (3.2)	0.54	27.7 (1.8)	27.4 (2.4)	27.4 (2.0)	27.6 (2.1)	0.87
REM SBDSQ (*n* = 274)	3.4 (2.0)	3.4 (2.4)	4.6 (3.2)	3.1 (2.2)	0.41	3.0 (2.4)	3.5 (2.5)	3.7 (3.0)	3.2 (2.8)	0.71
ESS (*n* = 274)	7.6 (4.9)	4.9 (3.1)	5.5 (2.6)	4.6 (2.1)	0.21	4.2 (2.7)	5.0 (3.6)	3.4 (3.5)	4.0 (2.5)	0.18
BDI-II (*n* = 274)	8.9 (4.4)	11.0 (7.4)	7.4 (6.8)	6.1 (5.6)	0.19	4.8 (6.8)	5.4 (4.8)	4.6 (9.3)	5.0 (6.4)	0.96
SCOPA-AUT (*n* = 274)	12.9 (5.6)	13.7 (7.3)	12.2 (9.0)	9.4 (5.4)	0.54	7.1 (4.5)	8.9 (5.9)	9.4 (10.6)	6.8 (5.5)	0.12
Parkinson Anxiety Scale (*n* = 274)	9.3 (6.8)	6.8 (7.0)	4.9 (3.8)	3.9 (4.4)	0.25	6.0 (6.1)	8.0 (7.5)	4.4 (7.8)	7.5 (7.8)	0.19

Values are mean (SD) for continuous variables and number (%) for categorical variables.

PD = Parkinson’s disease; MDS-UPDRS = Movement Disorder Society – Unified Parkinson Disease Rating Scale; MoCA = Montreal Cognitive Assessment; REM SBDSQ = Rapid Eye Movement Sleep Behavior Disorder Screening Questionnaire; ESS = Epworth Sleepiness Scale; BDI-II = Beck Depression Inventory-II; SCOPA-AUT = Scales for Outcomes in Parkinson’s Disease – Autonomic Dysfunction.

a Matching symbols indicate statistically significant pairwise differences (p < 0.05).

* Return of individual research results directly to the participant was not offered and was only provided upon request.

## Discussion

In a prospective, remote, natural history study of *LRRK2* G2019S carriers with and without PD, most participants were interested in the return of IRRs.

Interest was higher among those with PD (87%) versus those without PD (59%). We speculate that this interest gap may be due to differences in established care teams and the greater potential for results to induce anxiety or psychological distress among those genetically at risk for PD. Participants with and without PD were equally likely to request sharing with a PCP (33% with PD, 26% without PD) or themselves (38% with PD, 39% without PD). Participants without PD were less likely to request sharing with a neurologist (70% with PD, 6% without PD) likely reflecting low rates of neurological care in this group. Despite not directly asking if participants wanted to personally receive their IRRs, 39% of participants requested them. We speculate that a higher proportion would have opted to receive them if we had directly asked as we did with the return of IRRs to participants’ clinicians. In a prior remote observational study of individuals with and without PD, participants were asked if they wanted to receive their IRRs and 98.5% of participants opted into receiving their IRRs [7].

Prior studies that examined disclosure of genetic status among those with and at risk for PD in research settings found high satisfaction rates and low rates of adverse psychological experiences [8,9]. However, in the present study, participants were already aware of their genetic status at the time of enrollment, and the results disclosure focused on the presence of symptoms and signs of motor, cognitive, sleep, and mood disorders. Little is known about participant experiences with the disclosure of motor, cognitive, sleep, and mood assessment results among a genetically-at-risk population. The presence of motor signs, sleep dysfunction, depressive symptoms, or cognitive signs might raise concern among participants for the presence of PD, and it is possible that participants genetically at risk for PD had concerns about the psychological impact of results disclosure. We did not capture information on the reasons behind participants’ decisions for or against the return of IRRs. However, we found that among those without PD, those who requested sharing of IRRs with a clinician had higher scores on a patient-reported measure of motor symptoms, the MDS-UPDRS part II, than those who did not request any return of IRRs. We did not identify between group differences in examination-assessed MDS-UPDRS part III motor scores. Nonetheless, this suggests that individuals without PD with subtle motor symptoms may have been concerned about the presence of early PD and more interested in the return of IRRs.

We did not allow participants to decide what specific information was shared with each provider, which may have impacted their decision to share with their medical teams. However, other studies suggest the need for and importance of a personalized approach to the process of the return of IRRs. In a large survey study (*n* = 2,549), interest in IRRs was high (79%), yet 20% of respondents ranked basic information about themselves (e.g., lab tests, survey results) as the least valuable IRR, and this is the type of information we provided in VALOR-PD [1]. In the Project Baseline Health Study, which is a large, prospective, observational study of individuals with and at risk for different diseases [10], a majority of survey respondents (70%) cited learning more about their disease risk as a potential benefit of IRRs yet there was heterogeneity among participants regarding the desired type and quantity of information [2]. In VALOR-PD, the clinical information that we provide participants does not clearly inform disease risk. However, as the science evolves and we develop a better understanding of factors that modulate the risk of PD among *LRRK2* G2019S carriers and of preventative strategies, we may be able to offer more clearly actionable results to participants.

While this study has provided evidence that individuals with and genetically at risk for PD are interested in the return of IRRs and provides a framework for the return of clinical results, the study had limitations. One, while participants were located across the USA, more than 95% of study participants were white with at least some college education. Additionally, participants in this study were perhaps favorably predisposed to sharing IRRs as they had already sought out direct-to-consumer genetic testing through 23andMe and opted into receiving information about PD genetic variants. Therefore, our results may not be generalizable to other genetic cohorts or to traditional in-person cohorts. Two, as this study was not designed to assess the impact of the return of IRRs, we did not capture information regarding whether the return of IRRs was associated with a change in care, in the frequency of healthcare visits, or in health behaviors. More work is needed to inform best practices regarding the return of IRRs and understand the impact of the return of IRRs. With planned changes in VALOR-PD activities, we hope to expand the IRR summary, capture participant satisfaction with the IRR summary, and capture changes in health behaviors related to the IRR summary.

In conclusion, the majority of *LRRK2* G2019S participants in a remote natural history study were interested in the return of IRRs. More research is needed to understand the experiences of participants with the return of IRRs and to inform best practices for the return of IRRs.

## Data Availability

The de-identified data supporting the findings of this study are available on request from the corresponding author. The data are not publicly available due to privacy or ethical restrictions.
